# BCI Training Effects on Chronic Stroke Correlate with Functional Reorganization in Motor-Related Regions: A Concurrent EEG and fMRI Study

**DOI:** 10.3390/brainsci11010056

**Published:** 2021-01-06

**Authors:** Kai Yuan, Cheng Chen, Xin Wang, Winnie Chiu-wing Chu, Raymond Kai-yu Tong

**Affiliations:** 1 Department of Biomedical Engineering, The Chinese University of Hong Kong, Shatin, Hong Kong; kaiyuan@link.cuhk.edu.hk (K.Y.); chen_cheng@link.cuhk.edu.hk (C.C.); wangxin@link.cuhk.edu.hk (X.W.); 2 Department of Imaging and Interventional Radiology, The Chinese University of Hong Kong, Shatin, Hong Kong; winniechu@cuhk.edu.hk

**Keywords:** brain computer interface, stroke, robot hand training, EEG, fMRI, functional connectivity, effective connectivity

## Abstract

Brain–computer interface (BCI)-guided robot-assisted training strategy has been increasingly applied to stroke rehabilitation, while few studies have investigated the neuroplasticity change and functional reorganization after intervention from multimodality neuroimaging perspective. The present study aims to investigate the hemodynamic and electrophysical changes induced by BCI training using functional magnetic resonance imaging (fMRI) and electroencephalography (EEG) respectively, as well as the relationship between the neurological changes and motor function improvement. Fourteen chronic stroke subjects received 20 sessions of BCI-guided robot hand training. Simultaneous EEG and fMRI data were acquired before and immediately after the intervention. Seed-based functional connectivity for resting-state fMRI data and effective connectivity analysis for EEG were processed to reveal the neuroplasticity changes and interaction between different brain regions. Moreover, the relationship among motor function improvement, hemodynamic changes, and electrophysical changes derived from the two neuroimaging modalities was also investigated. This work suggested that (a) significant motor function improvement could be obtained after BCI training therapy, (b) training effect significantly correlated with functional connectivity change between ipsilesional M1 (iM1) and contralesional Brodmann area 6 (including *premotor area* (cPMA) and *supplementary motor area* (SMA)) derived from fMRI, (c) training effect significantly correlated with information flow change from cPMA to iM1 and strongly correlated with information flow change from SMA to iM1 derived from EEG, and (d) consistency of fMRI and EEG results illustrated by the correlation between functional connectivity change and information flow change. Our study showed changes in the brain after the BCI training therapy from chronic stroke survivors and provided a better understanding of neural mechanisms, especially the interaction among motor-related brain regions during stroke recovery. Besides, our finding demonstrated the feasibility and consistency of combining multiple neuroimaging modalities to investigate the neuroplasticity change.

## 1. Introduction

The most common and widely recognized impairment caused by stroke is motor impairment, which can be regarded as a loss of function in muscle control or movement or a limitation in mobility [1]. Neuroplasticity is a term describing the property of human brain to adapt to environmental pressure, experiences, and challenges including brain damage [2]. Various novel stroke rehabilitative methods for motor recovery, such as robotic therapies and noninvasive brain stimulation, have been developed based on basic science and clinical studies characterizing brain remodeling due to neuroplasticity [3,4]. BCI technology has been used for rehabilitation after stroke for years [5]. The majority of these studies are case reports of patients who operated a BCI to control either rehabilitation robots [6,7] or functional electrical stimulation (FES) [8,9]. BCI implies learning to modify the neuronal activity through progressive practice with contingent feedback and reward [10]. Therefore, changes in functional cortical activation patterns could remain when performing similar tasks as the BCI training even after the completion of the therapy [11]. In spite of some promising results achieved so far, BCI stroke rehabilitation is still a young field where different works report variable clinical outcomes [12]. The necessary functional connectivity changes among brain regions induced in stroke patients with lasting recovery effect remain unclear, with only several putative mechanisms been proposed [13].

Functional connectivity (FC), which measures the temporal correlation of the blood oxygen level-dependent (BOLD) signal between different regions at rest, has emerged as a powerful tool to map the functional organization of the brain. Studies imply that different behavioral impairments following stroke are related to the disruptions of communication in distributed brain networks with corresponding particular behavioral domains [14]. Furthermore, there is a growing awareness that disrupted functional interactions, especially inter-hemispheric interactions, are highly correlated with motor behavioral deficits and post-stroke recovery [15,16,17]. Although correlation-based connectivity using fMRI can investigate the relationship between distant brain regions, this kind of connectivity cannot provide any precise information related to the directionality of the information flow due to its poor time resolution. EEG is a prominent tool that offers a more direct measure of the electrophysiological signal with higher time resolution to explore the dynamic brain processes [18]. EEG signal can be regarded as the integration of all concurrently active sources in the brain [19]. Effective connectivity developed from Granger causality theory can be derived from EEG signal. This kind of connectivity reveals the directed information flow from one region to another and embeds both correlation and directional information between brain areas [20]. Among measures estimating effective connectivity, generalized partial directed coherence (GPDC) has shown good performance and is able to diminish the influence of noise [21]. To date, few studies focused on utilizing the effective connectivity change to characterize or predict the rehabilitation training effect for stroke subjects and meanwhile, multiple neuroimaging modalities were seldom combined simultaneously to investigate the brain recovery mechanism induced by training therapy.

In this study, two neuroimaging modalities—EEG and fMRI—were employed to evaluate the neuroplasticity changes in chronic stroke subjects after interventions using BCI robot hand training paradigm. Motor function of the paretic upper limb of stroke subjects was evaluated at three time points: before, immediately after, and 6 months after the interventions. Seed-based functional connectivity from resting-state fMRI and effective connectivity analysis from EEG were investigated to reveal the changes in neuroplasticity and interaction between different brain regions. Moreover, we also investigated the correlation between connectivity changes in two neuroimaging modalities and motor function changes after interventions. In the end, we explored the relationship between hemodynamic changes revealed by fMRI data and electrophysical changes revealed by EEG data. These findings may provide us with a better understanding of neural mechanisms during stroke recovery from BCI training and with guidance to future experimental design for upper limb.

## 2. Materials and Methods

### 2.1. Subject

Fourteen chronic stroke patients (13 males, mean age = 54 ± 8 years) with the right (n = 9) or left (n = 5) hemisphere impaired were recruited from local community. The inclusion criteria were (1) first-ever stroke, (2) onset of stroke diagnose more than 6 months, (3) a single unilateral brain lesion, (4) Hong Kong Montreal Cognitive Assessment (HK-MoCA) [22] score ≥22 to ensure sufficient cognitive function to understand instructions and perform tasks, (5) moderate to severe motor dysfunctions for the paretic upper extremity (Fugl–Meyer Assessment score for upper-extremity <47) [23], and (6) no additional rehabilitation therapies applied to the patient. Exclusion criteria were (1) aphasia, neglect, and apraxia, history of alcohol, drug abuse, or epilepsy; (2) severe hand spasticity; (3) hand deformity and wound; (4) bilateral infarcts; uncontrolled medical problems; and (5) serious cognitive deficits.

**Figure 1:**
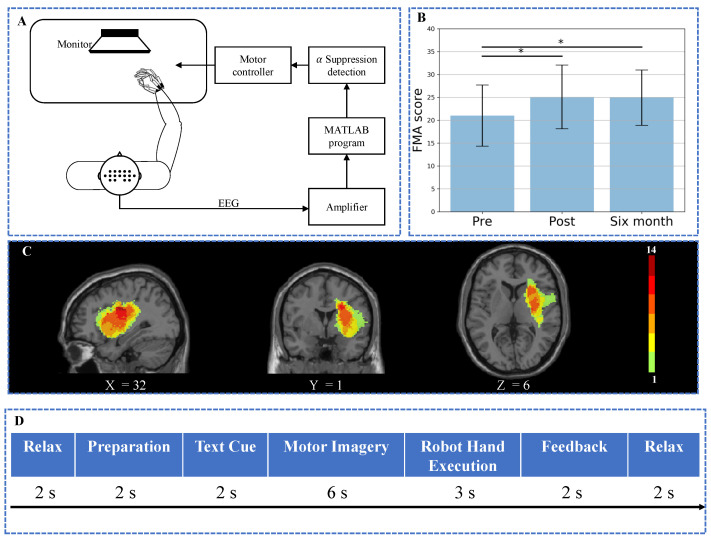
Illustration of the experimental settings and characteristics of subjects. (**A**) Schematic diagram of the whole training system. (**B**) Comparison of FMA score among pre-training, post-training, and six-month follow-up. Significant changes were seen in pre-training versus post-training and pre-training versus six-month follow-up. Error bars are standard errors. * p<0.05. (**C**) Lesion distribution of stroke subjects. The color bar represents the number of patients with lesions in the corresponding areas. The annotation of X, Y, Z represented the coordinate of the slice in Montreal Neurological Institute (MNI) space. (**D**) The sequence of training paradigm.

Motor functions of the paretic upper limbs for all stroke subjects were assessed with Fugl–Meyer Assessment for upper extremity (FMA) at three time points: before (Pre), immediately after (Post), and 6 months after (Six-month) the intervention respectively. Table 1 summarizes the demographics and clinical properties of the stroke subjects. The lesion distribution of the subjects is shown in Figure 1C. This study was approved by the Joint Chinese University of Hong Kong-New Territories East Cluster Clinical Research Ethics Committee. All subjects gave written consent before the intervention. This study was registered at https://clinicaltrials.gov (NCT02323061).

### 2.2. BCI Motor Imagery Training System

A BCI motor training system was designed as shown in Figure 1A [24]. A paradigm with fixed sequence showing instructions for motor imagery was played to guide the subject to complete a training task (shown in Figure 1D). An exoskeleton robot hand [25] was used to assist the paretic hand to accomplish grasp or open tasks. From fully extended position to fully flexed position, the fingers assembly provided 55 degrees and 65 degrees range of motion (ROM) for the MCP and PIP finger joints, respectively. During each trial, the subjects were asked to relax for 2 s followed by a white cross for 2 s to remind the subjects to do preparation. A text cue of “hand grasp” or “hand open” was then displayed for 2 s to illustrate the following motion. After that, the subjects were instructed to conduct motor imagery while a video clip with a duration of 6 s was displayed simultaneously for guidance. The trigger to the robot hand was sent based on the α suppression of EEG signal during the motor imagery to assist the subjects to complete grasp/open task in the following 3 s. Afterward, the α suppression score as the feedback was displayed for 2 s on the screen after robot hand execution to guide the subjects to achieve higher scores in the following trials. Finally, a 2 second rest was given to the subjects. Trials were repeated with grasping and opening tasks appeared alternately.

During the training, sixteen EEG electrodes (g.LADYbird, g.Tec Medical Engineering GmbH, Schiedlberg, Austria) were placed over the central area according to the international 10–20 system. EEG signals were referenced to the unilateral earlobe, grounded at the location of Fpz, and sampled at 256 Hz. The sampling signals were further processed in real-time using a band-pass filter (2–60 Hz) and a notch filter (48–52 Hz) to remove artifacts and power line noise, respectively. All electrodes were filled properly with conductive gel to ensure all the impedances were kept below 5 kΩ. All channels were used to generate the real-time topography of the brain dynamic potential for surveillance. Alpha suppression reflects an event-related desynchronization (ERD) of the EEG caused by an increase in neural activity [26], which has been widely utilized in BCI training field and has promising outcome [27,28]. To compute α suppression, the C3 or C4 channel was chosen according to the subject’s lesion side. The EEG data were transferred to frequency domain by a fast Fourier transform with a Hanning window, covering the EEG data during the motor imagery period (6 s) in the paradigm. The mean power in the α band (8–13 Hz) of the selected electrode was calculated. Then, the α suppression score was calculated using the following Equation [29],

(1) $$\begin{array}{c} \alpha_{ss}=-\frac{P_{MI}-P_{rest}}{P_{rest}}\times 100 \end{array}$$

where $\alpha_{ss}$ stands for the α suppression score, $P_{MI}$ stands for the α power of EEG during motor imagery, and $P_{rest}$ stands for the α power of EEG during resting state. The robot hand was triggered to apply a mechanical force to help hand grasp or open if the α suppression score is more than 20, which means the ratio of α power between motor imagery and rest was below 80% according to the average results of healthy subjects [30].

### 2.3. Interventional Protocols

All subjects received a 20-session BCI robot hand training with an intensity of 3–5 sessions per week. The whole training process was completed within 5–7 weeks. At the beginning of each training session, the subject was asked to sit in a height-adjustable chair and the trainer would standardize the posture to keep his/her shoulder naturally flexed and abducted, elbow flexed at 90 degrees, and arm pronated but with the wrist positioned neutrally without any flexion or extension. The subjects were instructed to try to maintain the standard posture during the whole training. During each session, 100 repetitive hand movements were performed by each subject with intermittent rest after every 10 trials. A robot hand was used to provide mechanical support to assist the subject in completing hand grasp or open task. Subjects were asked to imagine either grasping or releasing a cup following the instruction. Robot hand was triggered to help hand open or grasp if α suppression score calculated from real-time EEG signals was above 20 at $\alpha_{SS}$. All subjects were instructed to imagine the same movement with the affected hand during the stimuli display. The subjects would be reminded to conduct motor imagery if evidence of attempting movements was observed. The EMG activity was also monitored by the trainers. The experimental sequences for the training paradigm were developed based on Psychophysics Toolbox 3.0 (http://psychtoolbox.org/).

### 2.4. MRI and EEG Data Acquisition

MRI scans were acquired for all subjects before and after the intervention. A 3T Philips MR scanner (Achieva TX, Philips Medical System, Best, The Netherlands) with an 8-channel head coil was used to acquire high-resolution T1-weighted anatomical images (TR/TE = 7.47/3.45 ms, flip angle = 8${}^{\circ }$, 308 slices, voxel size = 0.6 × 1.042 × 1.042 mm${}^{3}$) using a T1-TFE sequence (ultrafast spoiled gradient echo pulse sequence), and BOLD fMRI images (TR/TE = 2000/30 ms, flip angle = 70${}^{\circ }$, 37 slices/volume, voxel size = 2.8 × 2.8 × 3.5 mm${}^{3}$) using a FE-EPI sequence (gradient echo echo-planar imaging sequence). The sequences were displayed using EPrime 2.0 (Psychology Software Tools, Sharpsburg, PA, USA). When acquiring resting-state fMRI, subjects were presented with a white crosshair in black background and instructed to rest while focusing on the fixation cross during the fMRI acquisition. The resting-state fMRI acquisition lasted for 8 min. The EEG data were acquired simultaneously with the fMRI using Neuroscan system (SynAmps2, Neuroscan Inc., Herndon, VA, USA). The MR-compatible EEG cap was placed with 64-channel Ag/AgCl EEG electrodes according to standard 10–20 system, as well as 2 extra electrocardiogram (ECG; left lower and near-midline upper chest) electrodes and 1 electrooculogram (EOG; below right eye) electrode. All recording impedances were kept below 5 kΩ. The reference channel was located at the point between Cz and CPz; AFz electrode was treated as ground. Signals were filtered between 0.1 and 256 Hz using an analog filter and sampled at 1000 Hz for off-line processing.

### 2.5. MRI Data Analysis

#### 2.5.1. MRI Data Preprocessing

The resting-state fMRI data were preprocessed using the Data Processing Assistant for Resting-State fMRI (DPARSF) toolbox [31] based on Statistical Parametric Mapping (SPM8) (http://www.fil.ion.ucl.ac.uk/spm). The details of MRI data preprocessing could be found in the Supplementary Materials. To perform the group statistical analysis later, subjects with left-hemispheric lesions were flipped along the midsagittal plane using MRIcron (www.mccauslandcenter.sc.edu/mricro/mricron), so that the lesions of all subjects were in the right hemisphere.

#### 2.5.2. Seed-Based FC Analysis

**Figure 2:**
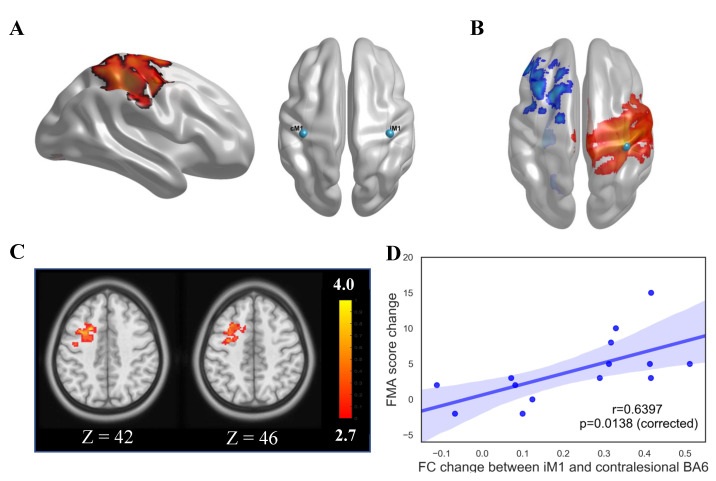
Seed selection and FC analysis result. (**A**) The color-coded area in the ipsilesional side of brain was significantly activated when subjects were doing the task according to task-based fMRI analysis. The right part demonstrated the locations of iM1 and cM1 seeds. The coordinate of iM1 and cM1 was at (38, −22, 56) and (−38, −22, 56), respectively, in Montreal Neurological Institute (MNI) space. (**B**) The color-coded area showed the FC map in the Pre session. (**C**) Functional connectivity between contralesional BA6 and iM1 seed was significantly changed. The color bar represents Z score. The white numbers aside the images represent the coordinate in MNI space. (**D**) Significant correlation was found between FMA score changes and functional connectivity change.

In order to avoid the bias induced by a prior determination of seeds based on a hypothesis or prior results, seed locations were decided based on task-fMRI. The details of defining the seed locations could be found in the Supplementary Materials. As a result, the ipsilesional M1 (iM1) seed location was set at (38, −22, 56) and contralesional M1 (cM1) was set at (−38, −22, 56) in Montreal Neurological Institute (MNI) space (Figure 2A). The average time course of the BOLD signal within the seeds during each resting-state scan was calculated and used as the regressors of interest in a subject-level general linear model (GLM) to assess the FC of each ROI with every other voxel in the brain. The seeds were checked one subject by one subject to ensure that they did not contain any lesioned voxels. This analysis produced maps of all voxels that were positively or negatively correlated with a seeds’ mean time courses. A demonstration of the group-level FC map when the seed was set at iM1 is shown in Figure 2B. After calculating individual seed-based correlation maps, a paired *t*-test was performed for each seed to see whether there were significant changes in FC brought by the training effect. Corrections for multiple comparisons at the cluster level were carried out using Gaussian random field theory (minimum Z>2.7; cluster-wise significance: P<0.05, corrected). To correct for multiple seeds, we only considered the clusters that had a probability greater than P=0.05/2 (2 was the number of seeds) as significant clusters [32]. All the analysis for mixed effect model and paired *t*-test were carried out in DPARSF toolbox [31]. We also explored whether the changes in functional connectivity in the brain were correlated with the assessment score changes. Pearson correlation coefficients were calculated between FMA score changes and the FC changes between seed area (ipsilesional M1) and corresponding areas (contralesional BA6 area) with which the FC has significantly changed (see Figure 2C) after 20 sessions of robot hand training.

### 2.6. EEG Analysis

#### 2.6.1. EEG Preprocessing

EEG data were processed with EEGLAB [33], Fieldtrip toolbox [34], Matlab signal processing toolbox (Mathworks, Natick, MA, USA), and custom-made codes. Standard preprocessing methods were adopted mainly to remove artifacts induced by MRI machine, inherent physiological artifacts [35] as well as other sources related to eye movement, muscle contraction and so on. The details could be found in the Supplementary Materials. For group-level analysis convenience, all EEG for those patients with left-hemisphere lesion was left-right flipped before signal processing procedure.

#### 2.6.2. Effective Connectivity Analysis

To extract the directional information transformation between regions, generalized partial directed coherence (GPDC), a multivariate (MV) approach based on Granger causality [36], was adopted. It was developed to circumvent the numerical problem related to time series scaling and is more robust for less clean signal [37]. Basically, GPDC is derived from the MV autoregressive (MVAR) model [38] using an appropriate order to fit the time series data. The MVAR model is described as

(2) $$\begin{array}{c} X\left(n\right)=\sum_{k=1}^{p}A\left(k\right)X\left(n-k\right)+W\left(n\right) \end{array}$$

where X is the time series, *p* is the model order, *k* is the time lag, A(k) is the fitting coefficient given time lag *k*, and W(n) is the residual white and uncorrelated noise. In our study, we used a MVAR model of order 10 corresponding to 40 ms of the signal to fit the EEG data, which is consistent with previous studies [39,40]. Leveraging the estimated model coefficients with the input noise variance into consideration, the value of GPDC can be calculated as

(3) $$\begin{array}{c} GPDC_{ij}\left(f\right)=\frac{\frac{1}{\sigma_{i}^{2}}{\left|A_{ij}\left(f\right)\right|}^{2}}{\sum_{m=1}^{M}\frac{1}{\sigma_{m}^{2}}{\left|A_{mj}\left(f\right)\right|}^{2}} \end{array}$$

where *f* is the frequency, $\sigma_{i}$ is the *i*th diagonal element of the residual noise covariance matrix, and $A_{ij}\left(f\right)$ is the coefficient transformed to the frequency domain which is related to interaction between the *i*th and *j*th signal.

GPDC can be treated as a measure of directional information or interaction from one signal to another with explicit directionality. Given frequency *f*, the *Information Flow (IF)* from $\Omega_{j}$ to $\Omega_{i}$ can be calculated as

(4) $$\begin{array}{c} IF_{ij}\left(f\right)=IF_{i\leftarrow j}\left(f\right)=\frac{\sum_{m\in \Omega_{i}}\sum_{n\in \Omega_{j}}GPDC_{mn}\left(f\right)}{N_{i}N_{j}} \end{array}$$

where Ω represents each ROI. $GPDC_{mn}$ indicates the GPDC value calculated between EEG signals from electrode *m* (in $\Omega_{i}$) and electrode *n* (in $\Omega_{j}$), respectively, with the direction from electrode *n* to electrode *m*. $N_{i}$ means the number of representative electrodes in $\Omega_{i}$. Given the direction in a pair of ROIs, ROI *i* and ROI *j* can be considered as *sink* region and *source* region of information flow, respectively, where sink region receives the signal or information flow sent from source region. Meanwhile, it can also be regarded that ROI *j* imposes causal influence on ROI *i*.

Based on the fMRI results, three regions of interest (ROIs) were determined for further effective connectivity analysis: contralesional premotor area (cPMA), supplementary motor area (SMA), and ipsilesional primary motor area (iM1). Representative EEG electrodes of the three ROIs were carefully selected, with *FC3* and *C3* for cPMA, *CZ* and *FCZ* for SMA, and *C4* for iM1 [41]. The values of information flow were calculated separately for each epoch and then averaged over all epochs [42]. In the frequency domain, we mainly focused on 8 to 30 Hz which contained two essential brain oscillatory activity frequency bands, alpha band (8–12 Hz) and beta band (12–30Hz) [43]. Furthermore, all information flow values were averaged across frequency bins. Pearson correlation coefficients were calculated between FMA score changes and the information flow change in both directions between cPMA and iM1 as well as between SMA and iM1 after training. Besides, the correlations between FC change (between iM1 and BA6 area) and corresponding information flow change were also investigated to inspect whether similar changes were associated between the findings from these two neuroimaging modalities.

### 2.7. Statistics

Statistic analyses were performed using SPSS 25.0 (IBM SPSS Statistics, Armonk, NY, USA) with the significance level set at p<0.05. A Friedman test at time level (Pre, Post, and Six-month) was applied to examine whether FMA score was changed after the intervention. Wilcoxon signed ranks test was used as post hoc test to examine significant changes of different combinations of three time points for FMA score. Pearson correlation coefficients were used to test the relationship between FMA score changes and the connectivity changes from two neuroimaging modalities. Bonferroni corrections were used for multiple comparisons.

## 3. Results

### 3.1. Results of Assessment Score

Friedman tests with time (Pre, Post, and Six-month) as within-subject factor indicated that significant effect of time was observed for FMA score ($\chi^{2}\left(2\right)=10.706$, p=0.005). Post hoc Wilcoxon signed ranks tests for FMA score indicated that there were significant increases in FMA scores between Pre and Post (Z=−2.846, p=0.004) as well as between Pre and Six-month (Z=−2.422, p=0.015), no significant change was found between Post and Six-month (Z=−0.153, p=0.878). The result was illustrated in Figure 1B. The results indicated that BCI robot hand training was able to promote motor recovery with a six-month long-term effect.

### 3.2. Results of Seed-Based FC Analysis

For the functional connectivity analysis with seed ROI at iM1, paired *t*-test showed that significant clusters were observed in contralesional Brodmann area 6 (BA6: *premotor cortex* and *supplementary motor area*). This suggested that the functional connectivity between iM1 and contralesional BA6 was significantly increased after 20 sessions of BCI robot hand training (Figure 2C). Pearson correlation analysis further revealed that the FC changes between iM1 and contralesional BA6 was significantly correlated with the FMA score changes (r=0.64, p=0.013, Figure 2D) after BCI robot hand training. There was no significant cluster survived after multiple comparison correction for the paired *t*-test when the seed ROI was at cM1.

### 3.3. Results of Information Flow and Correlation Analysis

**Figure 3:**
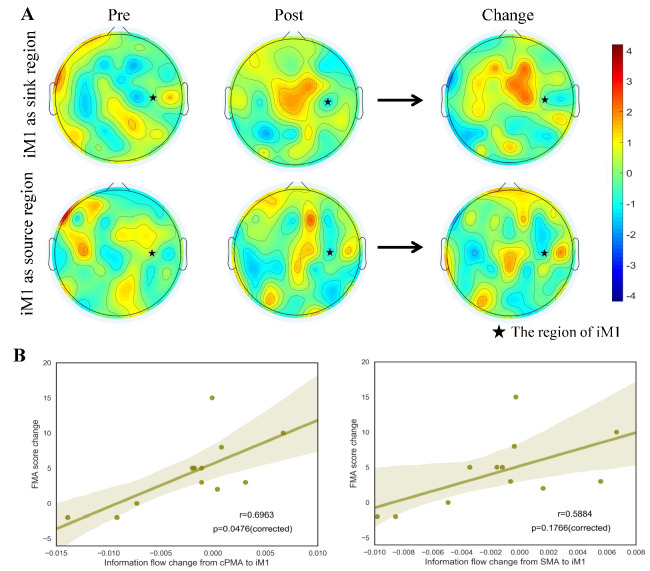
(**A**) The topography plots of information flow when iM1 set as sink/source regions. The first row represents the iM1 as sink region, where left and middle parts represent the condition of pre- and post-training, respectively. The right part is the change of information flow after training given iM1 as the sink region. The second row represents the iM1 as the source region, where left and middle parts represent the condition of pre- and post-training, respectively. All values were z-normalized across the whole brain for better visualization. (**B**) The correlation between FMA score changes and information flow change from cPMA to iM1 (left), SMA to iM1 (right).

Due to the lack of accurate fMRI triggers, the EEG signals of two subjects were not further analyzed to avoid processing bias, resulting in 12 subjects with both EEG and fMRI scans. Therefore, EEG analysis was only conducted on the remaining 12 subjects. First of all, we investigated the topography of the information flow change when iM1 was treated as either the *sink* or *source* regions. Figure 3A illustrates the two conditions (iM1 as *sink*/*source* region), where both premotor area and supplementary motor area showed notably changed patterns after the intervention. This phenomenon was more obvious when iM1 acted as the receiver (*sink* region) of the information flow.

Then, we explored the relationship between training effect and information flow changes when iM1 was treated as a sink region (i.e., information flow from cPMA or SMA to iM1). Pearson correlation analysis revealed that information flow change from cPMA to iM1 significantly correlated with the FMA score change after BCI robot hand training (r=0.6963, p=0.0476, Bonferroni corrected). The information flow change from SMA to iM1 correlated strongly with FMA score change (r=0.6046, p=0.1492, Bonferroni corrected, p=0.0373, uncorrected), although not significantly. The results are illustrated in Figure 3B. However, from the opposite direction, neither information flow change from iM1 to cPMA (r=−0.405, p=0.7660, Bonferroni corrected) nor from iM1 to SMA (r=0.1189, p=1, Bonferroni corrected) correlated significantly with FMA score change. The results are illustrated in the Supplementary Materials.

**Figure 4:**
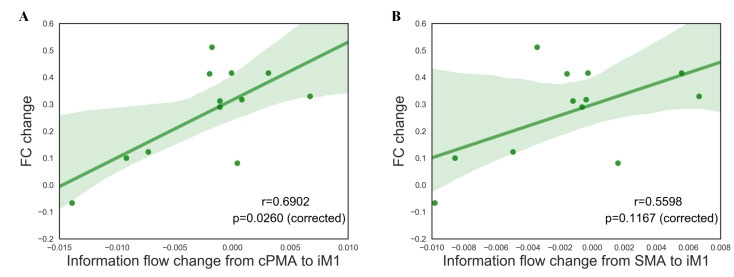
The correlation between functional connectivity change and information flow change from (**A**) cPMA to iM1, (**B**) SMA to iM1.

Upon determining the sink region role of iM1 in predicting training effect, we involved the FC changes from fMRI into correlation analysis. As illustrated in Figure 4, there is a significant association between the information flow change from cPMA to iM1 and their FC change (r=0.6902, p=0.0260, Bonferroni-corrected) as well as a strong relationship between the information flow change from SMA to iM1 and FC change (r=0.5859, p=0.1167, Bonferroni-corrected).

## 4. Discussion

This study investigated the neuroplasticity change and functional reorganization after BCI-guided robot-assisted training intervention from a multimodality neuroimaging perspective. Besides, the relationship between the neurological changes and motor function improvement was also investigated. Our study showed the change in the brain after BCI training therapy for chronic stroke survivors and provided a better understanding of neural mechanisms, especially the interaction among motor-related brain regions during stroke recovery using MRI and EEG. The correlation finding demonstrated the feasibility and consistency of combining multiple neuroimaging modalities to investigate the neuroplasticity change. Furthermore, our findings can provide insights into the design of stroke rehabilitation therapies for clinical application.

Noninvasive BCI systems have been introduced for upper limb rehabilitation after stroke for years, coupling with other interventions like occupational/physical therapy. Some studies reported significant motor function improvement after training with the help of BCI [12,44]. Biasiucci et al. illustrated that BCI coupled with FES elicited significantly better motor recovery than sham FES in chronic stroke patients. Furthermore, the underlying mechanism might relate to the FC between ipsilesional motor areas from neuroplasticity perspective. Kim et al. also claimed that combined action observational training (AOT) plus BCI-based FES (BCI-FES) with conventional therapy is more effective than sole conventional therapy in terms of upper limb motor improvement. Our study integrated brain–computer interface and robot hand, which further resembled such findings by exhibiting a significant training effect after 20 sessions of BCI robot hand training in chronic stroke. We noted that around 43% of subjects whose FMA improvement exceeded minimally clinical important difference (MCID) [45], which is 4.25. For chronic stroke subjects, undertaking significant motor recovery is very challenging. Peak neurological recovery from stroke occurs within the first one to three months and a number of studies have shown that recovery may continue at a slower pace after 6 months, with only 5% of patients continuing to recover for up to one year [46]. The rate of 43% in this study is comparable with other studies, such as 29% in [47] and 39% in [48]. The feedback offered by BCI facilitates the appraisal of performance by enforcing the sensory aspect in the sensorimotor loop [49]. External devices like robotic hand could provide haptic as well as proprioceptive feedbacks on the intended movement, thereby restoring the “action–perception coupling”. In this way, BCI robot-assisted training has already been shown to induce neuroplasticity [50]. The longitudinal training effect as well as the possible underlying neurophysiological mechanisms explored by fMRI and EEG were our main focus to evaluate the BCI training effect.

Recent findings indicate an association between resting-state FC and upper extremity control in persons with stroke [16]. Besides, FC changes were found in the sensorimotor network after motor learning [51,52]. FC could also serve as a biomarker of motor function recovery in stroke [53], which depicts the functional organization of the brain and neuroplasticity. In order to explore the FC changes within the sensorimotor network, we set two seeds at bilateral primary motor cortex, respectively. Consistent with previous studies, our study also showed a significant modulation of the FC between ipsilesional M1 and contralesional BA6 (premotor cortex and supplementary motor area). This neurological change was further highly correlated with behavioral changes (FMA score). The significant correlation between motor function improvement and FC change indicated that iM1 and contralesional BA6 could be regarded as the related areas to facilitate upper limb motor recovery after stroke. Some studies indicate that decreased inter-hemispheric connectivity between homologous areas tends to return to normal in patients who recovered well, whereas in poorly recovered patients, the degree of decreased interhemispheric connectivity correlates with motor function [16,54]. Our results further validate that interhemispheric FC changes within the sensorimotor network could be an indicator of motor recovery in chronic stroke subjects.

Given the low temporal resolution of fMRI, EEG could be another compensation perspective to capture the neuronal activity and dynamic brain processes more precisely. Recent findings illustrated that coherence with the primary motor area (M1) of the dominant hemisphere was a strong predictor of motor skill acquisition and thus could provide more information than that predicted by baseline behavior and demographics [55]. Besides, more and more studies focused on the effective connectivity, which could provide directional information compared with coherence-related coupling measurements [56]. Similar to previous studies, we found the influence (characterized by information flow defined in GPDC) from cPMA to iM1 significantly correlated with motor recovery. Meanwhile, a strong but not significant correlation between the influence from SMA to iM1 and recovery was also observed. Considering the findings from previous studies that stroke patients obtained weaker effective connection from cPMA to iM1 during motor imagery and execution compared with healthy subjects [57], our result validated this point from another perspective by showing that the enhanced effective interaction from cPMA to iM1 implied a better rehabilitation recovery. This might be partially due to the motor imagery procedure of the training therapy as the premotor cortex has been identified as the key node of motor imagery [58]. Our findings additionally suggest that effective connectivity from SMA to iM1 may also be a potential indicator for better recovery. This is not beyond our expectation as, together with cPMA, these areas are well known to be involved in planning, initiation and execution of motor commands [59]. It is worth noting that effective connectivity methods (e.g., GPDC) can provide concrete information of the active directed physical links between structures [60]. From this point, repairment or enhancement of these physical connections between motor-related regions should be crucial for recovery, which provides insights into therapy design for stroke rehabilitation. Combined with the result from fMRI, we could find that such concurrent collected EEG could provide more complementary information and disentangle the directionality of the FC extracted from fMRI. On the other hand, the significant correlation between FC change and directed information flow change within cPMA as well as SMA and iM1 also implied the inherent connection between these two neuroimaging modalities, which is in line with previous studies [61,62].

The structural base underlying the motor improvement and functional changes is also worthy of being investigated. Studies have found that the residual structural integrity of stroke subjects would also affect the outcome of training paradigms [63,64]. Diffusion tensor imaging (DTI) is a neuroimaging technique that allows for the quantitative assessment of white matter tract integrity [65]. Besides the removal of motion or eddy current artifacts, recent studies have also stated that it is also necessary to eliminate systematic errors in DTI using techniques such as b-matrix spatial distribution DTI technique (BSD-DTI) [66,67,68]. Studies have also interpreted that training could induce structural changes in the brain [69,70]. One study has indicated that motor improvement after BCI-guided training in stroke subjects correlated with corticospinal and transcallosal fiber changes assessed by DTI [70]. Therefore, changes in structural tracts in the lesioned brain after training would be an additional confirmation of the physiological changes indicated by EEG and fMRI. In the future study, structural images such as DTI might also be included to validate the findings in the physiological changes indicated by the functional neuroimaging. Due to the lack of a control group, we cannot affirmatively conclude that the observed significant association between FMA improvement and neuroplastic change was solely induced by our training intervention. It might also be related to placebo effect. Although under normal circumstances, it is quite difficult for chronic stroke patients to achieve comparable improvement without external intervention, a patient control group without real BCI-guided robot training will still be needed to exclude the placebo effect. Another confounding factor is about the observation of the correlation between changes in EEG and fMRI, which might not exclusively exist in the process of rehabilitation for stroke patients. It could also be observed in healthy subjects if they undergo the same training process, which might be the consequence of the reaction to the fact of the experiment. Hereby, a healthy control group will be needed to clarify this confusion in the future study. Besides, the sample size was not large, which might limit the generalization power. More patients should be recruited to validate and extend the findings of this study.

## 5. Conclusions

In summary, we investigated the relationship between the motor improvement induced by BCI robot-hand training and functional reorganization from hemodynamic together with electrophysical changes in motor-related regions through concurrent EEG and fMRI. We observed that the training effect significantly correlated with functional connectivity change between ipsilesional M1 and contralesional Brodmann area 6 derived from resting-state fMRI. Moreover, significant correlation was observed between motor improvement and information flow changes underlying crucial motor-related areas from EEG. Meanwhile, the corresponding changes from EEG and fMRI also illustrated significant relevance, which might implicate the inherent connection between these two neuroimaging modalities. We believe that the observed results indicate a positive effect of BCI robot-hand training and related neuroplastic changes in the brain, as evidenced by the observed correlations of EEG and fMRI parameters. However, due to the small number of stroke subjects and the lack of control groups, the experimental design could be further enhanced in the future to further validate the findings.

## Figures and Tables

**Table 1:** Demographics and clinical properties of the participants.

			Stroke				FMA (Max Score: 66)		
	Age		Onset	Lesion		Stroke			
No.	Range	Gender	(Years)	Side	Lesion Location	Type	Pre	Post	Six Month
S1	45–49	M	1	R	MFG, SFG, precentral,	I	19	34	28
					supramarginal, SMA				
S2	65–69	M	8	L	insula, putamen, IFG,	H	22	27	32
					temporal pole			
S3	65–69	M	1	R	insula, ITG, IOG,	H	13	16	27
					putamen				
S4	60–64	M	3	R	insula, putamen, IFG	I	16	14	18
					rolandic operculum				
S5	45–49	M	0.7	R	ITG, MTG, STG, MOG,	H	17	25	25
					angular, supramarginal				
S6	60–64	M	11	L	PLIC, putamen,	I	22	24	24
					insula, postcentral, SFG				
S7	55–59	M	6	R	insula, IFG	I	13	23	20
					rolandic operculum				
S8 ${}^{†}$	40–44	M	5	R	insula, rolandic operculum, IFG,	H	15	17	16
					STG, putamen, temporal pole				
S9	50–54	F	3	L	insula, rolandic operculum,	H	34	34	37
					putamen				
S10 ${}^{†}$	40–44	M	3	R	insula, MTG, STG, temporal pole,	H	17	20	20
					putamen, rolandic operculum				
S11	55–59	M	5	L	insula, IFG, putamen	H	28	33	24
S12	50–54	M	1	L	putamen, caudate nucleus	I	24	22	22
S13	55–59	M	7	R	putamen, temporal pole, IFG,	I	20	25	21
					insula, rolandic operculum				
S14	45–49	M	1	R	insula, putamen	H	34	37	35
mean ± std							21 ± 6.7	25 ± 7	25 ± 6

Abbreviations: F = female; FMA = Fugel–Meyer Assessment for upper limb; IFG = Inferior frontal gyrus; IOG = Inferior occipital gyrus; ITG = Inferior temporal gyrus; L = left hemisphere lesion; M = male; MFG = Middle frontal gyrus; MOG = Middle occipital gyrus; MTG = Middle temporal gyrus; PLIC = Posterior limb of the internal capsule; SFG = superior frontal gyrus; SMA = Supplementary motor area; STG = Superior temporal gyrus; R = right hemisphere lesion; I = ischemic; H = hemorrhage. ${}^{†}$ The EEG of these two subjects were not further analyzed.

## Data Availability

The data presented in this study are available on proper request from the corresponding author.

## Supplementary Materials

The following are available at https://www.mdpi.com/2076-3425/11/1/56/s1, Figure S1. The correlation between functional connectivity change and information flow change from iM1 to cPMA (left), iM1 to SMA (right).
