# Supplementary material for: BCI Training Effects on Chronic Stroke Correlate with Functional Reorganization in Motor-Related Regions: A Concurrent EEG and fMRI Study
Source: Brain Sci. 2021 Jan 6;11(1):56. doi: 10.3390/brainsci11010056 (PMC7824842; doi:10.3390/brainsci11010056)
Supplement: Supplementary file 1 [file brainsci-11-00056-s001.pdf]

# Supplementary Materials: BCI training effects on chronic stroke correlate with functional reorganization in motor-related regions: A concurrent EEG and fMRI study

**Kai Yuan, Cheng Chen, Xin Wang, Winnie Chiu-wing Chu and Raymond Kai-yu Tong**

## 1. FMRI Data Preprocessing

The first 10 volumes were discarded to assure the remaining volumes of fMRI data were at magnetization steady state. The remaining volumes were corrected with slice timing and realigned for head motion correction. Nuisance variables were then regressed out including white matter, cerebrospinal fluid (CSF), global mean signal and Fristion 24 head motion parameters [1]. To further control for head motion, scrubbing process were done for the volumes with framewise displacement (FD) value execeed 0.3 [2]. Then the anatomical dataset was aligned to the functional dataset. Detrending and temporal band-pass filtering (0.01 Hz - 0.1 Hz) [3] were performed to remove higher frequency physiological noise and lower frequency scanner drift. Subsequently, the functional images were spatially normalized to the Montreal Neurological Institute (MNI) template (MNI152: average T1 brain image constructed from 152 normal subjects), resliced to 2 mm $\times$ 2 mm $\times$ 2 mm voxels, and smoothed with a Gaussian kernel with a full-width at half-maximum (FWHM) of 6 mm.

## 2. Defining the Seed Locations

When acquiring task-based fMRI, subjects were asked to try to do grasp and open using their paretic hand when a mark of "L" or "R" (decided by each subject's paretic hand) appeared on the screen and were also asked to maintain 6 seconds until the mark disappeared from the screen. An event-related design was adopted and randomized time intervals from 14 to 20 seconds were assigned between every two tasks. Two 6-minute task-based fMRI runs were performed for each subject.

The task-based fMRI data were also preprocessed using DPARSF toolbox. Similar preprocessing steps were performed on task-based fMRI data except that the threshold for FD value was set to 0.7 in the motion scrubbing step [4] and no band-pass filter was used. Subjects with left-hemispheric lesions were also flipped along the midsagittal plane. The preprocessed task-based fMRI data were fitted into a GLM for subject-level analysis where each of the events were convolved using a canonical hemodynamic response function (HRF) and used as a regressor. Besides, six motion regressors were also included in the design matrix to regress out motion-related fluctuations in the BOLD signal. As a result, each subject acquired a $t$-map at each session. The statistical maps from all sessions were used to do the group level analysis (one sample $t$-test). Voxels were identified as significantly active if they surpassed a threshold of $z > 2.7$ and corrected using Gaussian random field theory at a threshold of $p < 0.05$ at a cluster level. The seed locations for iM1 and cM1 were defined based on the group-level activation map.

## 3. EEG Data Preprocessing

Under the condition where MRI was acquired simultaneously, the switching of magnetic field gradients would pollute and overwhelm EEG signal which led to low signal to noise ratio (SNR). A principle component analysis (PCA)-based optimal basis set (OBS) algorithm [5] was adopted to remove the MRI gradient artifact and the onset markers indicating the beginning of each fMRI volume, generated by MRI scanner were also provided for better extraction and selection of artifactual features. The output EEG signal were double-checked visually to ensure that the amplitude was not grandiosely large. The time course of heartbeat artifact was determined with a R-peak detection algorithm [6]. The final ECG artifact was eliminated channel-wisely using the strategy which combined

independent component analysis, OBS and an information-theoretic rejection criterion developed by Liu and colleagues [6].

After that, EEG signal was band-pass filtered from 2 to 40 Hz using a Butterworth non-causal filter. Subsequently, bad channels were removed and reconstructed using spherical spline interpolation with neighbor electrodes. Following that, all data were common average referenced. According to the fMRI trigger markers, these data were segmented into non-overlapping two-second epochs where the first and last several data segments were removed due to signal instability. Bad epochs were rejected based on statistical measurement metrics (e.g. z-score, variance, min, and max etc.) with remaining ones further inspected visually to guarantee the signal quality. We utilized adaptive mixture independent component analysis (AMICA) algorithm [7] to separate EEG signals into spatially static and maximally temporally independent components [8]. The components related to residual artifact induced by MRI scanning, Electrooculogram (EOG) artifact and muscular artifact were rejected. Processes of remaining components were then projected back to all original channels. Finally, we applied a surface Laplace filter with the spherical spline method [9] to increase the topographical selectivity, eliminate the volume conduction and highlight the high-spatial-frequency components while attenuating low ones [10].

## 4. Correlation of Information Flow When iM1 Was the Source Region

Then we explored the relationship between training effect and information flow changes when iM1 was treated as a source region (i.e. information flow from iM1 to cPMA or SMA). However, neither information flow change from iM1 to cPMA ($r = -0.405$, $p = 0.7660$, Bonferroni corrected) nor from iM1 to SMA ($r = 0.1189$, $p = 1$, Bonferroni corrected) correlated significantly or strongly with FMA score change.

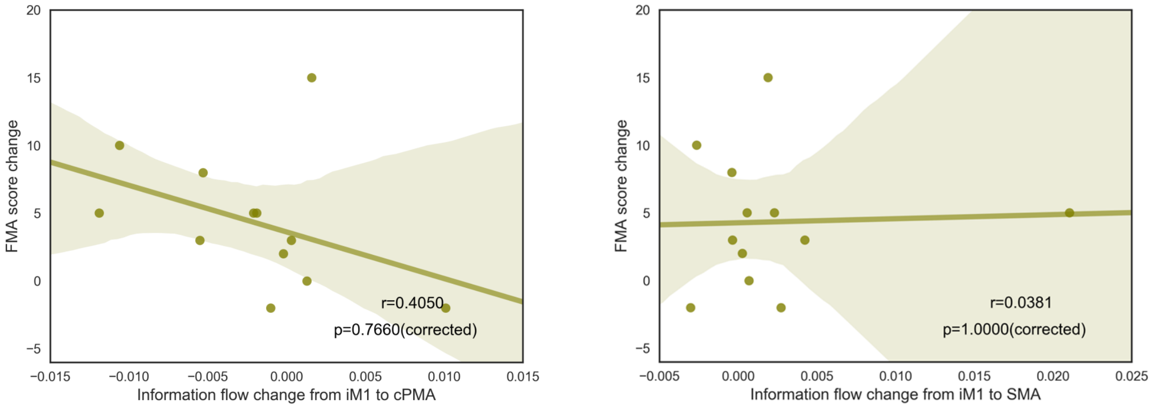


**Figure S1.** The correlation between functional connectivity change and information flow change from iM1 to cPMA (left), iM1 to SMA (right).

1. Friston, K.J.; Williams, S.; Howard, R.; Frackowiak, R.S.J.; Turner, R. Movement-Related effects in fMRI time-series. *Magnetic Resonance in Medicine* **1996**, *35*, 346–355. doi:10.1002/mrm.1910350312.
2. Power, J.D.; Barnes, K.A.; Snyder, A.Z.; Schlaggar, B.L.; Petersen, S.E. Spurious but systematic correlations in functional connectivity MRI networks arise from subject motion. *NeuroImage* **2012**, *59*, 2142 – 2154. doi:https://doi.org/10.1016/j.neuroimage.2011.10.018.
3. Auer, D.P. Spontaneous low-frequency blood oxygenation level-dependent fluctuations and functional connectivity analysis of the 'resting' brain. *Magnetic Resonance Imaging* **2008**, *26*, 1055 – 1064. Proceedings of the International School on Magnetic Resonance and Brain Function, doi:https://doi.org/10.1016/j.mri.2008.05.008.

4. Siegel, J.S.; Power, J.D.; Dubis, J.W.; Vogel, A.C.; Church, J.A.; Schlaggar, B.L.; Petersen, S.E. Statistical improvements in functional magnetic resonance imaging analyses produced by censoring high-motion data points. *Human Brain Mapping* **2014**, *35*, 1981–1996. doi:10.1002/hbm.22307.
5. Niazy, R.; Beckmann, C.; Iannetti, G.; Brady, J.; Smith, S. Removal of FMRI environment artifacts from EEG data using optimal basis sets. *NeuroImage* **2005**, *28*, 720 – 737. doi:https://doi.org/10.1016/j.neuroimage.2005.06.067.
6. Liu, Z.; de Zwart, J.A.; van Gelderen, P.; Kuo, L.W.; Duyn, J.H. Statistical feature extraction for artifact removal from concurrent fMRI-EEG recordings. *NeuroImage* **2012**, *59*, 2073 – 2087. doi:https://doi.org/10.1016/j.neuroimage.2011.10.042.
7. Palmer, J.A.; Kreutz-Delgado, K.; Makeig, S. Super-Gaussian Mixture Source Model for ICA. Independent Component Analysis and Blind Signal Separation; Rosca, J.; Erdogmus, D.; Príncipe, J.C.; Haykin, S., Eds.; Springer Berlin Heidelberg: Berlin, Heidelberg, 2006; pp. 854–861.
8. Makeig, S.; Bell, A.J.; Jung, T.P.; Sejnowski, T.J. Independent Component Analysis of Electroencephalographic Data. In *Advances in Neural Information Processing Systems 8*; Touretzky, D.S.; Mozer, M.C.; Hasselmo, M.E., Eds.; MIT Press, 1996; pp. 145–151.
9. Perrin, F.; Pernier, J.; Bertnard, O.; Giard, M.; Echallier, J. Mapping of scalp potentials by surface spline interpolation. *Electroencephalography and Clinical Neurophysiology* **1987**, *66*, 75 – 81. doi:https://doi.org/10.1016/0013-4694(87)90141-6.
10. Cohen, M.X. *Analyzing neural time series data: theory and practice*; Issues in clinical and cognitive neuropsychology, The MIT Press: Cambridge, Massachusetts, 2014.

**Publisher's Note:** MDPI stays neutral with regard to jurisdictional claims in published maps and institutional affiliations.
